# Serum Creatinine/Cystatin C Ratio as a Predictor of In-hospital Mortality in Patients Hospitalized with Acute Exacerbation of Chronic Obstructive Pulmonary Disease

**DOI:** 10.1007/s00408-022-00568-5

**Published:** 2022-09-15

**Authors:** Zhixiang Chen, Lei Zha, Xiao Ma, Jing Xu, Dandan Huang, Wenlong Wu, Long Chen, Feng Yang, Weiling Liao, Wenhua Wang

**Affiliations:** 1 Department of Respiratory and Critical Care Medicine, Wuhu Hospital of Traditional Chinese Medicine, No. 430, Jiuhua South Road, Wuhu, Anhui China; 2 Department of Emergency and Critical Care, Conch Hospital of Anhui Medical University, No. 327, Jiuhua South Road, Wuhu, Anhui China; 3 Institute of Infection and Global Health, University of Liverpool, Liverpool, UK; 4 Department of Respiratory and Critical Care Medicine, Postgraduate School of Wuhu Hospital of Traditional Chinese Medicine Affiliated With Anhui University of Traditional Chinese Medicine, No. 430, Jiuhua South Road, Wuhu, Anhui China

**Keywords:** Chronic obstructive pulmonary disease, Acute exacerbation, Serum creatinine/cystatin C ratio, Mortality

## Abstract

**Purpose:**

Low serum creatinine/cystatin C ratio (CCR) is associated with unfavorable characteristics in patients with chronic obstructive pulmonary disease (COPD); however, the relationship between CCR and in-hospital mortality of patients with acute exacerbation of COPD (AECOPD) is unexplored. Our objective was to assess the value of CCR for predicting in-hospital mortality of patients hospitalized with AECOPD.

**Methods:**

Patients with AECOPD (*n* = 597) were retrospectively enrolled. Patient’s clinical characteristics and laboratory tests, including serum cystatin C and creatinine, were reviewed. The prediction value of CCR was evaluated using area under the receiver operating characteristic curve (AUC) values. Factors potentially impacting in-hospital mortality were investigated using univariate and multivariate logistic regression analyses.

**Results:**

Mortality rate during hospitalization was 10.05%. CCR was lower in non-surviving vs. survived patients (41.67 vs. 61.52, *P* < 0.001). AUC value for CCR for in-hospital mortality prediction was 0.79 [95% confidence interval (CI) 0.73–0.85]. On multivariate logistic regression analysis, in-hospital mortality was strongly associated with CCR < 52.27 [odds ratio (OR) 6.23, 95% CI (3.00–12.92), *P* < 0.001], age ≥ 81 years [OR 2.97, 95% CI (1.20–7.37), *P* = 0.019], oxygenation index < 300 [OR 3.28, 95% CI (1.27–8.44), *P* = 0.014], CRP > 8 mg/L [OR 1.84, 95% CI (1.15–2.95), *P* = 0.012], and D-dimer > 500 ng/L [OR 5.19, 95% CI (1.51–17.79), *P* = 0.009].

**Conclusions:**

CCR was significantly lower, and is a potential prognostic indicator, in patients with AECOPD who died during hospitalization.

## Introduction

Chronic obstructive pulmonary disease (COPD) is an airway disease characterized by incompletely reversible airflow limitation that causes high mortality and morbidity worldwide [1]. Acute exacerbation (AE) is the most common reason for hospitalization among patients with COPD, and is associated with a rapid decline of respiratory function, impaired quality of life, and poor prognosis [2, 3]. In addition, frequent exacerbation is recognized as a significant risk factor contributing to increased mortality in patients with COPD [4]. Thus, early and accurate diagnosis of AE, risk stratification of patients, and prediction of treatment response warrant increased research attention.

Malnutrition and sarcopenia are common among patients with COPD, particularly when the disease progresses to an advanced stage [5, 6], and is associated with adverse effects on exercise, muscle function, lung function, and increased rates of exacerbation, hospitalization, and mortality, as well as higher costs [7–9]. Therefore, accurate assessment of malnutrition and sarcopenia in patients with COPD is of great importance [10]. Currently, the most commonly used methods to evaluate nutritional status in patients with COPD in the clinic are as follows: ultrasonography, including diaphragm thickness and echo intensity of rectus femoris; computed tomography (CT) scan, including cross-sectional areas of the midthigh and erector spinae muscles; and screening and assessment tools, including body mass index (BMI), the Mini Nutrition Assessment, Nutrition Risk Screening 2002, and Subjective Global Assessment [11]. However, these approaches have limitations in terms of specificity, sensitivity, and convenience [12, 13]; thus, there is an urgent need to find alternative evaluation methods. The serum creatinine (SCr)/Cystatin C (Cys C) ratio (CCR) has emerged as a promising surrogate marker of muscle wasting and dysfunction [14–16] independent of renal function [17] since its first proposal by Kashani et al. [18]. Further, CCR is a marker of poor prognosis in patients with coronary artery disease [19] and serious neurological disease [20], as well as in patients receiving intensive care and continuous kidney replacement therapy [21].

In the context of COPD, CCR is a valuable marker for evaluating skeletal muscle mass, disease severity, and prognosis. Specifically, CCR is a useful indicator of muscle strength decline in male patients with AECOPD [22], and is significantly associated with forced vital capacity and forced expiratory volume in 1 s (FEV1) values, particularly in former smokers [23]. Further, low CCR values are associated with longer duration of hospital admission, increased dyspnea, greater functional impairment in patients admitted for AECOPD [24], and is also an independent predictor of hospitalization in the following year for outpatients with stable COPD [25]. Nevertheless, to the best of our knowledge, there has been no published study that has assessed the relationship between CCR value and in-hospital mortality of patients hospitalized with AECOPD. Therefore, in this study, we aimed to clarify the potential value of the CCR for predicting in-hospital mortality of patients hospitalized with AECOPD.

## Materials and Methods

### Study Design and Subjects

Patients with AECOPD hospitalized at the Department of Respiratory and Critical Care Medicine of Wuhu Hospital of Traditional Chinese Medicine, China were enrolled in this retrospective observational study between January 2016 and February 2022. The primary outcome was in-hospital mortality. Diagnosis of COPD was based on the Global Initiative for Chronic Obstructive Lung Disease (GOLD 2018) criteria [1]. Inclusion criteria were hospitalized patients with AECOPD, defined as an acute deterioration in respiratory symptoms of sufficient severity to require admission. Exclusion criteria were as follows: (1) age < 40 years; (2) end-stage renal disease (ESRD), defined as estimated GFR < 15 mL/min/1.73 m^2^, or already receiving renal replacement therapy; (3) acute renal injury; (4) receiving palliative care; or (5) missing data required for the study. For patients with multiple hospital admissions during the study period, only the first admission record was used. As a retrospective analysis, the study was not registered.

### Data Collection

Clinical data were extracted from the electronic medical records system. Data collected included general information (age, gender, and number of hospitalizations due to AE in the previous year); underlying disease (hypertension, diabetes mellitus, coronary heart disease, lung cancer, and heart failure); laboratory tests [platelet count (PLT), SCr, Cys C, C-reactive protein (CRP), D-dimer, oxygenation index (OI) (SpO_2_/FiO_2_), partial pressure of carbon dioxide (pCO_2_), and blood lactate (Lac)]; and treatment outcomes (non-invasive ventilation (NIV), invasive mechanical ventilation (IMV), ICU admission, length of hospital stay (LOS), and prognosis (survival/death)). Hypercapnia was defined as a pCO_2_ > 45 mmHg. CCR was calculated as CCR = SCr/Cys C. Data were collected from laboratory tests conducted at the first examination on admission.

### Statistical Analysis

Quantitative variables are expressed as median with interquartile range (IQR), and qualitative variables as frequencies or percentages. Statistical comparisons of variables between the survived and non-survived groups were performed using the chi-square test for categorical variables and the Mann–Whitney *U* test for continuous variables. Receiver operating characteristic (ROC) curves were used to evaluate the ability of CCR to predict in-hospital mortality, and Youden’s index was used to determine the optimal cut-off point. The predictive accuracy of CCR was determined using the area under the ROC curve (AUC). Subsequently, patients were classified into two groups based on the CCR cut-off value. Risk factors potentially influencing in-hospital mortality were analyzed using univariable and multivariable logistic regressions. The goodness of fit of models was assessed using calibration curves. The cut-off values for OI, CRP, and D-dimer were determined based on the clinical reference intervals used in our hospital laboratory. Age was categorized based on tertile values for the cohort. All statistical analyses were performed using SPSS 26.0 (IBM, SPSS) and GraphPad Prism 8.0 (GraphPad Software).

## Results

### Comparison of Patient Characteristics Between Non-survival and Survival Groups

**Fig. 1:**
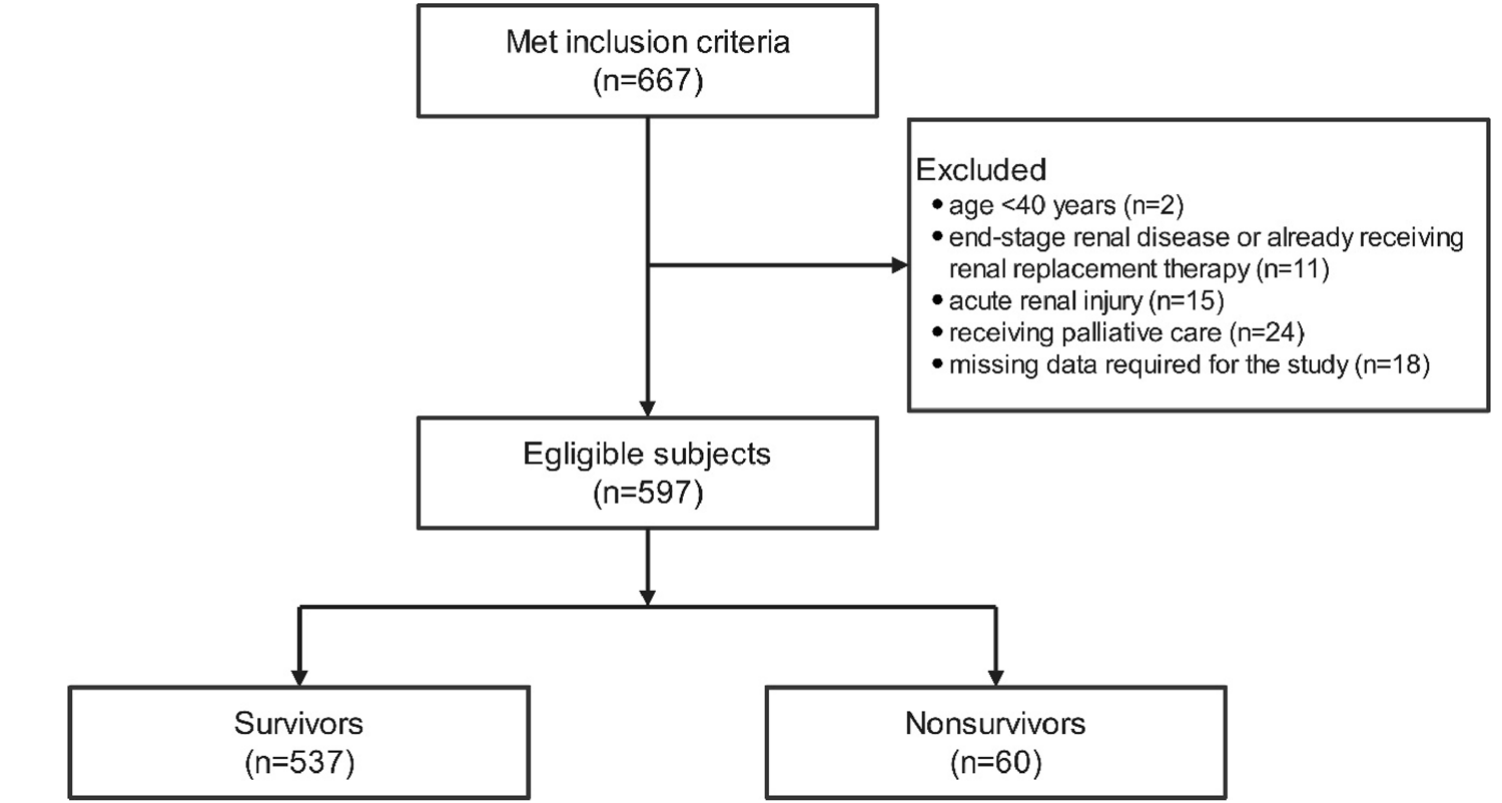
Flowchart of enrolled patients

A flow chart showing patient inclusion in this study is presented as Fig. 1. In total, 597 patients were ultimately included in this study and 60 (10.1%) died during hospitalization. Participant baseline characteristics and comparisons between non-survival and survival groups are summarized in Table 1. Median (IQR) CCR values in total, the non-survived, and survived patients were 59.7 (48.4–72.4), 41.7 (34.7–52.1), and 61.5 (50.8–73.8), respectively; the difference between survived and non-survived patients was significant (*P* < 0.001). However, SCr did not differ significantly between the two groups (*P* = 0.590). Compared to survived patients, those who died were older, had more frequent hospitalization due to AE in the previous year, longer LOS, and included higher proportions with heart failure, lung cancer, and coronary heart disease. CRP, Cys C, D-dimer levels, proportion with hypercapnia, and patients requiring NIV, IMV, and ICU admission were higher in the non-survivor group. Further, OI and Hb were significantly lower in non-survived than survived patients, whereas there were no significant differences in PLT, PDW, and Lac between the two groups.

**Table 1:** Demographic and clinical characteristics of overall participants and comparisons of non-survival and survival groups

Variable	Total	Survival	Non-survival	*P* value
Number of patients	597	537	60	
Age (years)	77.00 (71.00, 83.00)	76.00 (71.00, 82.00)	81.00 (77.00, 86.00)	< 0.001
Gender				0.048
Male	433 (72.5)	383 (71.3)	50 (83.3)	
Female	164 (27.5)	154 (28.7)	10 (16.7)	
Number of hospitalizations due to AE in the previous year	1.00 (0.00, 2.00)	1.00 (0.00, 2.00)	2.00 (2.00, 3.00)	< 0.001
Secondary outcome				
Hypercapnia	224 (37.5)	189 (35.2)	35 (58.3)	< 0.001
NIV	122 (20.4)	95 (17.7)	27 (45.0)	< 0.001
IMV	30 (5.0)	18 (3.4)	12 (20.0)	< 0.001
ICU admission	50 (8.4)	34 (6.3)	16 (26.7)	< 0.001
Length of stay (days)	10.00 (7.00, 15.00)	10.00 (7.00, 14.00)	20.50 (10.00, 35.00)	< 0.001
Comorbidity				
Diabetes mellitus	84 (14.1)	77 (14.3)	7 (11.7)	0.572
Hypertension	256 (42.9)	229 (42.6)	27 (45.0)	0.727
Lung cancer	17 (2.8)	10 (1.9)	7 (11.7)	< 0.001
Coronary heart disease	227 (38.0)	197 (36.7)	30 (50.0)	0.044
Heart failure	253 (42.4)	219 (40.8)	43 (71.7)	< 0.001
Laboratory examinations				
Oxygenation index	287.00 (214.10, 343.40)	296.30 (222.95, 346.85)	227.15 (164.90, 274.38)	< 0.001
pCO_2_ (mmHg)	42.70 (34.50, 57.85)	42.10 (34.40, 56.25)	50.95 (37.05, 68.50)	0.008
Lac (mmol/L)	1.10 (0.90, 1.50)	1.10 (0.90, 1.50)	1.10 (0.80, 1.70)	0.653
Hb (g/L)	125.00 (112.00, 138.00)	126.00 (113.00, 139.00)	111.00 (100.50, 131.00)	< 0.001
PLT (× 10^9^/L)	165.00 (125.00, 213.50)	164.00 (124.00, 211.00)	170.50 (127.25, 234.25)	0.341
PDW	13.40 (11.70, 16.20)	13.40 (11.80, 16.20)	13.50 (11.50, 15.10)	0.319
SCr (μmol/L)	75.30 (59.90, 98.25)	74.90 (60.60, 97.75)	81.15 (54.58, 122.55)	0.590
Cys C (mmol/L)	1.25 (1.00, 1.70)	1.23 (0.97, 1.56)	1.91 (1.29, 2.94)	< 0.001
CRP (mg/L)	14.85 (3.40, 60.75)	12.85 (3.20, 54.40)	51.10 (14.33, 148.45)	< 0.001
D-dimer (ng/L)	870.00 (450.00, 1700.00)	794.35 (420.00, 1848.50)	2070.00 (1099.25, 2901.25)	< 0.001
CCR	59.69 (48.40, 72.38)	61.52 (50.78, 73.83)	41.67 (34.70, 52.07)	< 0.001

Data are presented as n (%) or median (25th centile, 75th centile)

*AE* acute exacerbation, *Hypercapnia* pCO_2_ > 45 mmHg, *NIV* non-invasive ventilation, *IMV* invasive mechanical ventilation, *ICU* intensive care unit, *pCO2* partial pressure of carbon dioxide, *Lac* blood lactic acid, *Hb* hemoglobin, *PLT* platelets, *PDW* platelet distribution width, *SCr* serum creatinine, *Cys C* cystatin C, *CRP* C-reactive protein, *CCR* serum creatinine/cystatin C ratio

### Relationships Between CCR Levels and Clinically Relevant Results

**Fig. 2:**
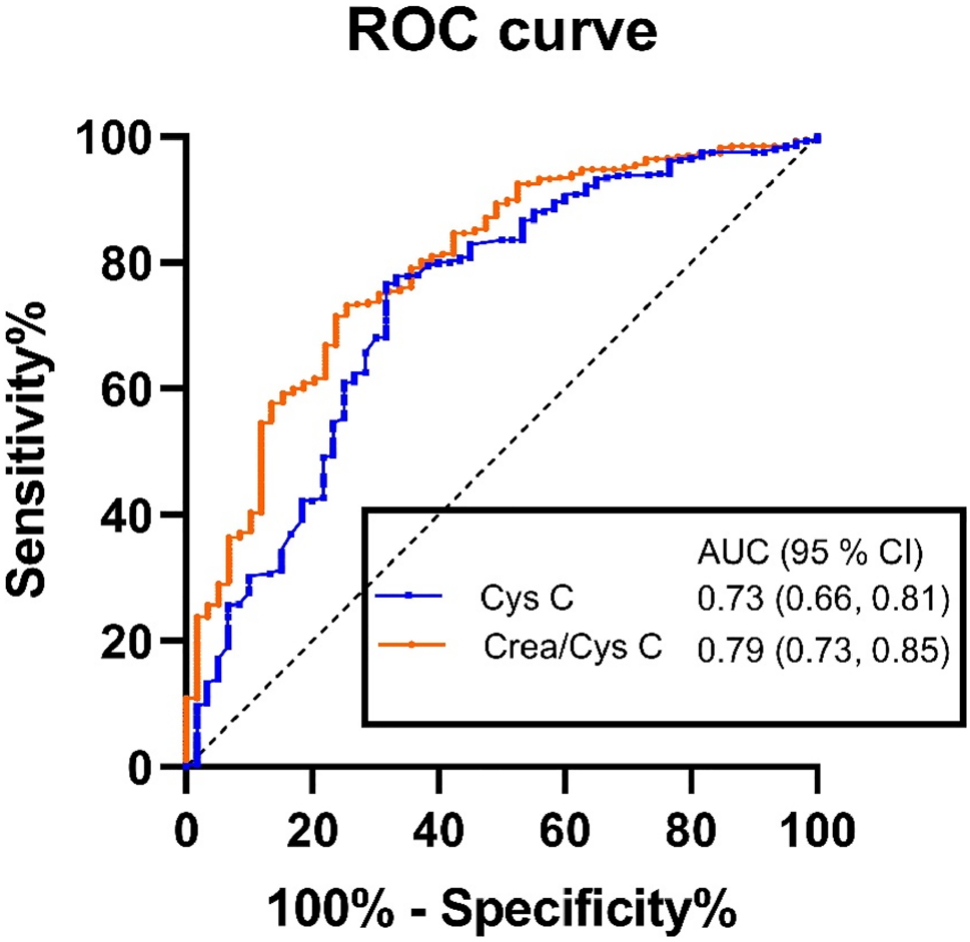
Receiver operating characteristic curves to evaluate the ability of CCR and Cys C to predict in-hospital mortality. *ROC* receiver operating characteristic, *AUC* area under the ROC curve, *Cys* C cystatin C, *SCr* serum creatinine, *CCR* serum creatinine/cystatin C ratio, *IQR* interquartile range

ROC curves to evaluate the ability of CCR and Cys C predicting in-hospital mortality are shown in Fig. 2. The AUC values of CCR and Cys C were 0.79 (95% CI 0.73–0.85) and 0.73 (95% CI 0.66–0.81), respectively. The optimal cut-off CCR value was 52.27, corresponding to sensitivity and specificity values of 76.7% and 71.5%, respectively. Next, we divided participants into low (CCR < 52.27, *N* = 199, 33.3%) and high (CCR ≥ 52.27, *N* = 398, 66.7%) CCR groups, according to the CCR cut-off value and found significant differences between the two groups (Table 2). Compared with the high CCR group, patients in the low CCR group were more likely to be older, have more frequent hospitalization due to AE in the previous year, and have a longer LOS, and included higher proportions with coexisting lung cancer and heart failure, ICU admission, and elevated D-dimer. Conversely, Hb levels were significantly lower in the low CCR group than those in the high group. Further, there were significantly more male patients in the high CCR group. No significant differences in OI, PLT, PDW, or CRP were observed between the groups.

**Table 2:** Patient characteristics stratified by serum creatinine/cystatin C ratio

Variable	CCR ≥ 52.27	CCR < 52.27	*P* value
Number of patients	398	199	
Age (years)	76.00 (71.00, 82.25)	78.00 (73.00, 83.00)	0.028
Gender			< 0.001
Male	312 (78.4)	121 (60.8)	
Female	86 (21.6)	78 (39.2)	
Prognosis			< 0.001
Survived	384 (96.5)	153 (76.9)	
Died	14 (3.5)	46 (23.1)	
Number of hospitalizations due to AE in the previous year	1.00 (0.00, 2.00)	1.00 (0.75, 2.00)	0.024
Secondary outcome			
Hypercapnia	143 (35.9)	81 (40.7)	0.256
NIV	76 (19.1)	46 (23.1)	0.251
IMV	19 (4.8)	11 (5.5)	0.691
ICU admission	27 (6.8)	23 (11.6)	0.047
Length of stay (days)	10.00 (7.00, 14.00)	11.00 (8.00, 19.00)	0.023
Comorbidity			
Diabetes mellitus	55 (13.8)	29 (14.6)	0.803
Hypertension	176 (44.2)	80 (40.2)	0.349
Lung cancer	5 (1.3)	12 (6.0)	0.001
Coronary heart disease	151 (37.9)	76 (38.2)	0.952
Heart failure	151 (37.9)	111 (55.8)	< 0.001
Laboratory examination			
Oxygenation index	291.25 (222.98, 346.83)	282.30 (193.00, 336.80)	0.067
pCO_2_ (mmHg)	42.25 (34.40, 56.73)	43.40 (35.30, 59.90)	0.211
Lac (mmol/L)	1.10 (0.90, 1.50)	1.10 (0.90, 1.50)	0.914
Hb (g/L)	128.00 (114.00, 140.00)	119.00 (106.00,134.00)	< 0.001
PLT (× 10^9^/L)	165.00 (127.00, 206.00)	162.00 (122.00, 224.00)	0.863
PDW	13.40 (11.63, 16.20)	13.50 (11.80, 16.00)	0.909
SCr (μmol/L)	79.65 (65.75, 102.95)	64.00 (51.30, 90.00)	< 0.001
Cys C (mmol/L)	1.16 (0.94, 1.48)	1.50 (1.20, 2.26)	< 0.001
CRP (mg/L)	14.20 (3.40, 60.50)	15.70 (3.20, 62.70)	0.515
D-dimer (ng/L)	810.00 (420.00, 1470.00)	1010.00 (510.00, 2110.00)	0.006

Data are presented as n (%) or median (25th centile, 75th centile)

*CCR* serum creatinine/cystatin C ratio, *AE* acute exacerbation, *Hypercapnia* pCO_2_ > 45 mmHg, *NIV* non-invasive ventilation, *IMV* invasive mechanical ventilation, *ICU* intensive care unit, *pCO2* partial pressure of carbon dioxide, *Lac* blood lactic acid, *Hb* hemoglobin, *PLT* platelets, *PDW* platelet distribution width, *SCr* serum creatinine, *Cys C* cystatin C, *CRP* C-reactive protein

### Univariate and Multivariate Regression Analyses of Factors Associated with In-hospital Mortality

**Fig. 3:**
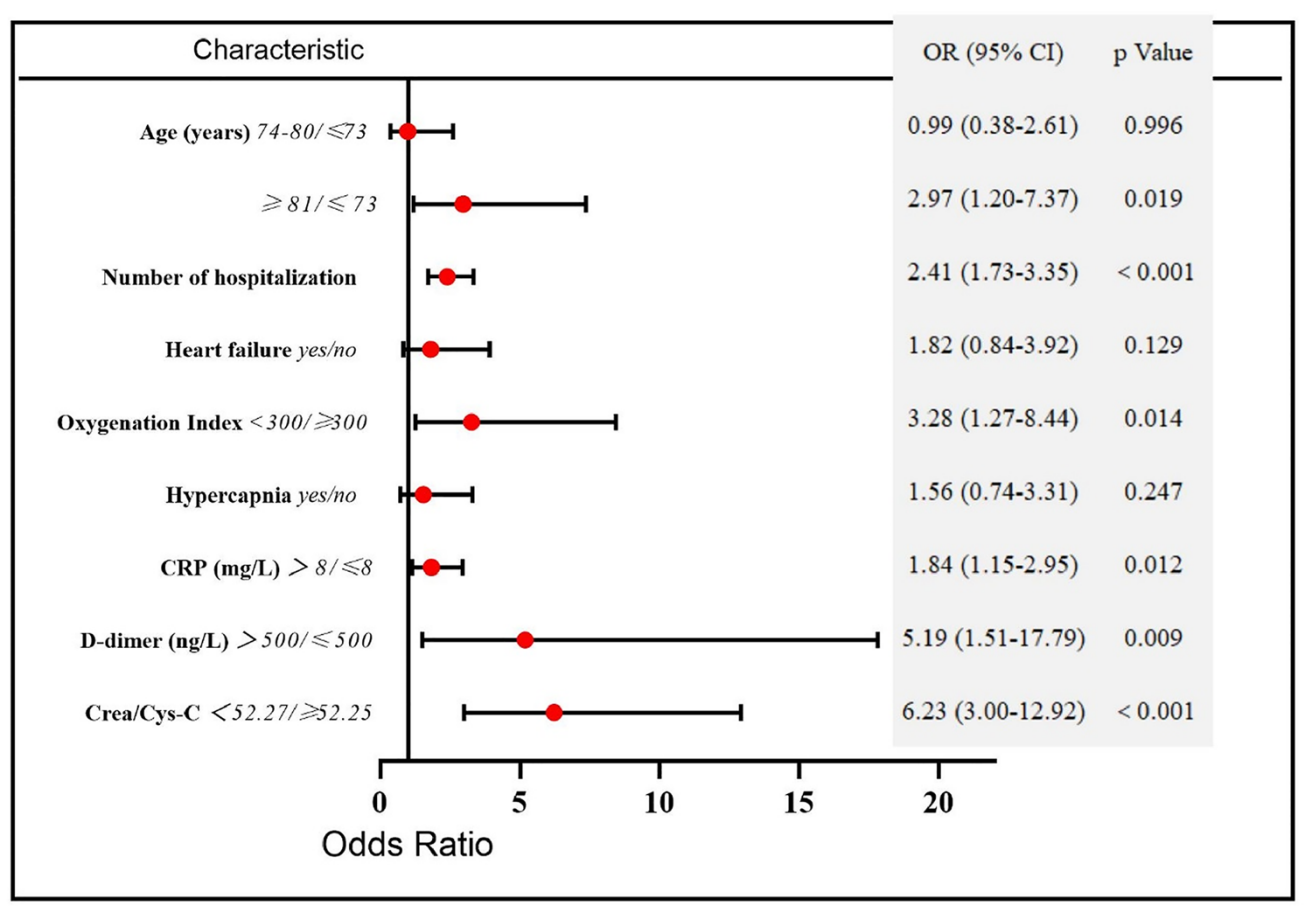
Multivariate logistic regression analysis and Forest plot. *Hypercapnia* pCO_2_ > 5 mmHg, *CRP* C-reactive protein, *CCR* serum creatinine/cystatin C ratio, *OR* odds ratio, *CI* confidence interval

The results of univariate logistic regression analysis of risk factors associated with in-hospital mortality are presented in Table 3. The in-hospital mortality of patients with low CCR levels was 23.1%, which was significantly higher than that in patients with high CCR levels (3.5%) [OR 8.2, 95% CI (4.4–15.4)]. Further, age, number of hospitalizations due to AE, hypercapnia, heart failure, OI, CRP, and D-dimer were also significant predictors of in-hospital mortality. Based on the literature, previous clinical experience, and the results of univariate analysis, we selected CCR value, age, number of hospitalizations due to AE in the past year, coexisting heart failure, OI, hypercapnia, CRP, and D-dimer for inclusion in multivariate logistic regression analysis (Fig. 3), which indicated that CCR < 52.27 [OR 6.23, 95% CI (3.00–12.92), *P* < 0.001], age ≥ 81 years, number of hospitalizations due to AE in the previous year, OI < 300, CRP > 8 mg/L, and D-dimer > 500 ng/L were significant predictors of in-hospital mortality for patients hospitalized with AECOPD. A calibration curve for the multivariable logistic regression model demonstrated that the model has sufficient goodness of fit (Hosmer‒Lemeshow goodness of fit test, *P* = 0.693).

**Table 3:** Univariate logistic regression analysis of in-hospital mortality between the survival and non-survival group

Variable	OR	95% CI	*P* value
Age (years)			0.002
74–80 vs. ≤ 73	1.660	0.750, 3.675	0.211
≥ 81 vs. ≤ 73	3.364	1.650, 6.860	0.001
Number of hospitalizations due to AE in the previous year	2.140	1.679, 2.728	< 0.001
Hypercapnia	2.578	1.498, 4.436	0.001
Heart failure	3.673	2.041, 6.608	< 0.001
Oxygenation index			< 0.001
< 300/ ≥ 300	6.011	2.802, 12.896	< 0.001
CRP (mg/L)	1.006	1.002, 1.009	0.001
> 8/ ≤ 8	3.190	1.570, 6.484	0.001
D-dimer (ng/L)			
> 500/ ≤ 500	6.264	2.235, 17.560	< 0.001
CCR			
< 52.27/ ≥ 52.27	8.246	4.406, 15.436	< 0.001

*Hypercapnia* pCO_2_ > 45 mmHg, *OR* odds ratio, *CI* confidence interval, *AE* acute exacerbation, *CRP* C-reactive protein, *CCR* serum creatinine/cystatin C ratio

## Discussion

In the present study, the value of CCR for predicting in-hospital mortality in patients hospitalized with AECOPD was assessed. We found that CCR was significantly lower in the non-survival group than in the survival group among patients hospitalized for AECOPD. The AUC of the ROC curve for CCR prediction of in-hospital mortality was 0.79 (95% CI 0.73–0.85), and the optimal cut-off CCR value was 52.27, with maximum sensitivity and specificity of 76.7% and 71.5%, respectively. More importantly, multivariate logistic regression analysis confirmed that CCR < 52.27 was an independent risk factor for in-hospital mortality after adjusting for confounding factors, including hypercapnia and history of heart failure. These results clearly indicate that the CCR value has potential to be an excellent predictor of short-term prognosis for patients with AECOPD during hospitalization. We also found that age ≥ 81 years, number of hospitalizations due to AE in the previous year, OI < 300, CRP > 8 mg/L, and D-dimer > 500 ng/L were strongly associated with increased hospital mortality.

Plasma Cys C has been used to predict the prognosis of diseases [26–28], including COPD. Hu et al. [29] reported that Cys C was a strong and independent risk factor for hospital mortality secondary to AECOPD. They reported an AUC value for prediction of death using Cys C of 0.77 (95% CI 0.70–0.84), which is generally consistent with the results of our study; however, importantly, the AUC for CCR prediction of in-hospital mortality in the current study was 0.79 (95% CI 0.73–0.85), which was higher than that for Cys C alone (0.73, 95% CI 0.66–0.81), indicating that CCR has better short-term predictive value.

In this study, patients in the high CCR group tended to be younger and more often male than those in the CCR low group. One possible explanation for this is that CCR value is strongly associated with muscle mass [30], and low CCR can be considered an independent predictor of sarcopenia [31]. In general, females have less muscle mass than males, and aging is also associated with decreased muscle mass [32]. Hence, CCR values tend to be lower in older adult and female populations [33]. We also found that patients in the low CCR group had more frequent hospitalization due to AE in the previous year than those in the CCR high group. This conclusion is supported by a similar finding reported by Hirai et al. [34], in which low CCR was a predictor of severe exacerbation, independent of age, COPD Assessment Test score, and %FEV1 (*P* < 0.01). Lu et al. [19] investigated the association between CCR and the prevalence of major adverse cardiovascular events in patients with coronary artery disease and found that those in the low CCR group were more likely to have congestive heart failure (*P* < 0.05). Jung et al. [35] found that patients with CCR values in the highest quartile had shorter lengths of ICU and hospital stay (both *P* < 0.001) in patients with cancer. These results are all concordant with our findings; however, no significant differences in the requirement for, or duration of, mechanical ventilation were identified between the high and low CCR groups in our study, inconsistent with the results of a previous investigation [20]; further studies are needed to explain this discrepancy.

One of the most important findings of our study is that CCR < 52.27 is significantly associated with in-hospital mortality for patients hospitalized with AECOPD [OR 6.23, 95% CI (3.00–12.92), *P* < 0.001], independently of hypercapnia and history of heart failure. This may be attributable to reduced muscle mass, which can be accurately assessed by a low CCR value [14, 34, 36], and is directly correlated with poor prognosis in various diseases, including short-term and long-term mortality. Jaitovich et al. [37] performed a prospective observational study to investigate the association of skeletal muscle and fat mass at ICU admission with survival and disability at hospital discharge. They concluded that pectoralis muscle area, determined by CT scan at the time of ICU admission, was associated with 6-month and hospital survival, as well as ICU-free days, despite not being a good predictor of regaining an independent lifestyle after discharge. Zheng et al. [38] demonstrated that lower CCR could be used to identify sarcopenia in patients with esophageal cancer and was associated with shorter overall survival. Further, they found the all-cause mortality risk gradually decreased with increased CCR. In addition to CCR < 52.27, we found that age ≥ 81 years, frequency of hospitalization due to AE in the previous year, OI < 300, CRP > 8 mg/L, and D-dimer > 500 ng/L were closely associated with in-hospital mortality. These findings are in line with the conclusions of earlier research. Crisafulli et al. [39] found that age and acute severe hypoxemia at admission were significant independent predictors of mortality in patients hospitalized with AECOPD. Soler-Cataluña et al. [40] showed that patients with three or more acute COPD exacerbations had the greatest mortality risk. Hu et al. [41] found that D-dimer was a strong and independent risk factor associated with in-hospital and 1-year death of patients with AECOPD requiring hospitalization. Sneh et al. [42] reported that high CRP levels at baseline, and persistently elevated CRP may predict mortality in patients with AE-COPD requiring mechanical ventilation.

Inevitably, this study has some inherent limitations. First, there may have been selection bias and the results are less reliable than those of a prospective study would be, because of the single-center retrospective design. Second, although patients enrolled in this study had confirmed COPD diagnosis, most had disease too severe to have lung function examined during hospitalization. Thus, we could not consider the impact of lung function indices. Third, the cut-off value of CCR was determined by the Youden index, which might not be a practical value in clinical practice to some extent. If the aim is to design a scoring tool, other practical techniques should be considered, as described in these studies [43, 44]. Finally, we did not include other indicators used to assess nutritional status, such as BMI, geriatric nutritional risk index, controlling nutritional status score, and prognostic nutritional index; therefore, we could not compare the predictive efficacy of these different indicators for in-hospital mortality.

## Conclusions

CCR was significantly lower among patients hospitalized with AECOPD who died during admission than those who survived; therefore, it could be a powerful predictor of mortality by incorporating it within clinical risk scores.
